# Supplementary material for: High-density linkage to physical mapping in a unique Tall × Dwarf coconut (Cocos nucifera L.) outbred F2 uncovers a major QTL for flowering time colocalized with the FLOWERING LOCUS T (FT)
Source: Front Plant Sci. 2024 Jun 3;15:1408239. doi: 10.3389/fpls.2024.1408239 (PMC11180721; doi:10.3389/fpls.2024.1408239)
Supplement: Supplementary File S7 — BLAST alignment results of the Arabidopsis thaliana putative flowering signals mediating protein FT (At1g65480) mRNA, complete cds against the YANG chromosome 3. [file DataSheet_1.pdf]

```
Query= AY065378.1 Arabidopsis thaliana putative flowering signals mediating
protein FT (At1g65480) mRNA, complete cds

Length=840

>Cocos nucifera cultivar Hainan Tall coconut chromosome 3, whole genome shotgun sequence
Sequence ID: CM017874.1 Length: 82374452
Range 1: 70554038 to 70554249

Score:108 bits(119), Expect:3e-23,
Identities:151/212(71%),  Gaps:0/212(0%), Strand: Plus/Plus

Query  392       GAGATTGTGTGTTACGAAAATCCAAGTCCCACTGCAGGAATTCATCGTGTCGTGTTTATA  451
                 ||||||||||| || || | |||| | ||  |    || || || ||  || | ||| |
Sbjct  70554038  GAGATTGTGTGCTATGAGAGTCCACGGCCGGCGCTTGGCATCCACCGGTTCATCTTTGTG  70554097

Query  452       TTGTTTCGACAGCTTGGCAGGCAAACAGTGTATGCACCAGGGTGGCGCCAGAACTTCAAC  511
                  |||| |  ||||||||  |||| ||||||||||| || ||||||||||| || ||| ||
Sbjct  70554098  CTGTTCCAGCAGCTTGGGCGGCAGACAGTGTATGCCCCTGGGTGGCGCCAAAATTTCGAC  70554157

Query  512       ACTCGCGAGTTTGCTGAGATCTACAATCTCGGCCTTCCCGTGGCCGCAGTTTTCTACAAT  571
                 || || || ||||| ||  ||||||| |||||    || || || ||||| |  |  ||
Sbjct  70554158  ACCCGGGACTTTGCAGAACTCTACAACCTCGGATCACCAGTCGCAGCAGTCTATTTTAAC  70554217

Query  572       TGTCAGAGGGAGAGTGGCTGCGGAGGAAGAAG  603
                 || ||||| |||   |||| ||| || |||||
Sbjct  70554218  TGCCAGAGAGAGTCGGGCTCCGGCGGGAGAAG  70554249



Range 2: 73265231 to 73265450

Score:88.7 bits(97), Expect:3e-17,
Identities:155/222(70%),  Gaps:3/222(1%), Strand: Plus/Plus
```

```
Query  392       GAGATTGTGTGTTACGAAAATCCAAGTCCCACTGCAGGAATTCATCGTGTCGTGTTTATA  451
                 ||||||||  |||| ||||  || |||||     |||| || || ||  | ||||||
Sbjct  73265231  GAGATTGTAGGTTATGAAAGCCCTAGTCCGGTGTCAGGGATCCACCGCATGGTGTTTGCG  73265290

Query  452       TTGTTTCGACAGCTTGGCAGGCAAACAGTGTATGCACCAGGGTGGCGCCAGAACTTCAAC  511
                  |||| | |||| | |||||  |||  |||| ||| |||| |  ||| |  |||||||||
Sbjct  73265291  CTGTTCCAACAGTTAGGCAGAGAAAGCGTGTTTGCCCCAGAGATGCGGCCCAACTTCAAC  73265350

Query  512       ACTCGCGAGTTTGC-TGAGATCTACAATCTCGGCCTTCCCGTGGCCGCAGTTTTCTACAA  570
                 ||  |  | |||||  | || | || |||| || |  || || ||||| || | || |||
Sbjct  73265351  ACCAGGAATTTTGCACGGGAAC-ACTATCTGGGGCCACCGGTTGCCGCTGTCTACTTCAA  73265409

Query  571       TTGTCAGAGGGAGAGTGGCTGCGGAGGAAGAAGACTTTAGAT  612
                 ||| ||||||||   ||||| ||| || |||||| || ||||
Sbjct  73265410  TTGCCAGAGGGAATCTGGCTCCGGCGGTAGAAGA-TTCAGAT  73265450
```
