# Supplementary material for: High-density linkage to physical mapping in a unique Tall × Dwarf coconut (Cocos nucifera L.) outbred F2 uncovers a major QTL for flowering time colocalized with the FLOWERING LOCUS T (FT)
Source: Front Plant Sci. 2024 Jun 3;15:1408239. doi: 10.3389/fpls.2024.1408239 (PMC11180721; doi:10.3389/fpls.2024.1408239)
Supplement: Supplementary File S8 — BLAST alignment results of the Arabidopsis thaliana putative flowering signals mediating protein FT (At1g65480) mRNA, complete cds against the WANG chromosome 4. [file DataSheet_2.pdf]

```
Query= AY065378.1 Arabidopsis thaliana putative flowering signals mediating
protein FT (At1g65480) mRNA, complete cds

>GWHBEBT00000004   OriSeqID=Chr04   Len=180429735
Length=180429735

 Score = 108 bits (119),  Expect = 7e-21
 Identities = 151/212 (71%), Gaps = 0/212 (0%)
 Strand=Plus/Minus

Query  392        GAGATTGTGTGTTACGAAAATCCAAGTCCCACTGCAGGAATTCATCGTGTCGTGTTTATA  451
                  ||||||||||| || || | |||| | ||  |    || || || ||  || | ||| |
Sbjct  164688301  GAGATTGTGTGCTATGAGAGTCCACGGCCGGCGCTTGGCATCCACCGGTTCATCTTTGTG  164688242

Query  452        TTGTTTCGACAGCTTGGCAGGCAAACAGTGTATGCACCAGGGTGGCGCCAGAACTTCAAC  511
                   |||| |  ||||||||  |||| ||||||||||| || ||||||||||| || ||| ||
Sbjct  164688241  CTGTTCCAGCAGCTTGGGCGGCAGACAGTGTATGCCCCTGGGTGGCGCCAAAATTTCGAC  164688182

Query  512        ACTCGCGAGTTTGCTGAGATCTACAATCTCGGCCTTCCCGTGGCCGCAGTTTTCTACAAT  571
                  || || || ||||| ||  ||||||| |||||    || || || ||||| |  |  ||
Sbjct  164688181  ACCCGGGACTTTGCAGAACTCTACAACCTCGGATCACCAGTCGCAGCAGTCTATTTTAAC  164688122

Query  572        TGTCAGAGGGAGAGTGGCTGCGGAGGAAGAAG  603
                  || ||||| |||   |||| ||| || |||||
Sbjct  164688121  TGCCAGAGAGAGTCGGGCTCCGGCGGGAGAAG  164688090


 Score = 88.7 bits (97),  Expect = 7e-15
 Identities = 155/222 (70%), Gaps = 3/222 (1%)
 Strand=Plus/Plus

Query  392        GAGATTGTGTGTTACGAAAATCCAAGTCCCACTGCAGGAATTCATCGTGTCGTGTTTATA  451
                  ||||||||  |||| ||||  || |||||     |||| || || ||  | ||||||
Sbjct  168065258  GAGATTGTAGGTTATGAAAGCCCTAGTCCGGTGTCAGGGATCCACCGCATGGTGTTTGCG  168065317
```

```
Query  452        TTGTTTCGACAGCTTGGCAGGCAAACAGTGTATGCACCAGGGTGGCGCCAGAACTTCAAC  511
                   |||| | |||| | |||||  |||  |||| ||| |||| |  ||| |  |||||||||
Sbjct  168065318  CTGTTCCAACAGTTAGGCAGAGAAAGCGTGTTTGCCCCAGAGATGCGGCCCAACTTCAAC  168065377

Query  512        ACTCGCGAGTTTGC-TGAGATCTACAATCTCGGCCTTCCCGTGGCCGCAGTTTTCTACAA  570
                  ||  |  | |||||  | || | || |||| || |  || || ||||| || | || |||
Sbjct  168065378  ACCAGGAATTTTGCACGGGAAC-ACTATCTGGGGCCACCGGTTGCCGCTGTCTACTTCAA  168065436

Query  571        TTGTCAGAGGGAGAGTGGCTGCGGAGGAAGAAGACTTTAGAT  612
                  ||| ||||||||   ||||| ||| || |||||| || ||||
Sbjct  168065437  TTGCCAGAGGGAATCTGGCTCCGGCGGTAGAAGA-TTCAGAT  168065477
```
